# Gauze Detection and Segmentation in Minimally Invasive Surgery Video Using Convolutional Neural Networks

**DOI:** 10.3390/s22145180

**Published:** 2022-07-11

**Authors:** Guillermo Sánchez-Brizuela, Francisco-Javier Santos-Criado, Daniel Sanz-Gobernado, Eusebio de la Fuente-López, Juan-Carlos Fraile, Javier Pérez-Turiel, Ana Cisnal

**Affiliations:** 1 Instituto de las Tecnologías Avanzadas de la Producción (ITAP), Universidad de Valladolid, Paseo del Cauce 59, 47011 Valladolid, Spain; danisago2@gmail.com (D.S.-G.); efuente@uva.es (E.d.l.F.-L.); jcfraile@eii.uva.es (J.-C.F.); turiel@eii.uva.es (J.P.-T.); ana.cisnal@uva.es (A.C.); 2 Escuela Técnica Superior de Ingenieros Industriales, Universidad Politécnica de Madrid, Calle de José Gutiérrez Abascal, 2, 28006 Madrid, Spain; javiersantoscriado@hotmail.com

**Keywords:** convolutional neural networks, image segmentation, image object detection, surgical tool detection, minimally invasive surgery

## Abstract

Medical instruments detection in laparoscopic video has been carried out to increase the autonomy of surgical robots, evaluate skills or index recordings. However, it has not been extended to surgical gauzes. Gauzes can provide valuable information to numerous tasks in the operating room, but the lack of an annotated dataset has hampered its research. In this article, we present a segmentation dataset with 4003 hand-labelled frames from laparoscopic video. To prove the dataset potential, we analyzed several baselines: detection using YOLOv3, coarse segmentation, and segmentation with a U-Net. Our results show that YOLOv3 can be executed in real time but provides a modest recall. Coarse segmentation presents satisfactory results but lacks inference speed. Finally, the U-Net baseline achieves a good speed-quality compromise running above 30 FPS while obtaining an IoU of 0.85. The accuracy reached by U-Net and its execution speed demonstrate that precise and real-time gauze segmentation can be achieved, training convolutional neural networks on the proposed dataset.

## 1. Introduction

Laparoscopic surgery is the minimally invasive alternative to open surgery of the abdomen. Two or more surgical instruments, along with an endoscope that allows the patient to be observed internally, are introduced into the abdominal cavity through several small incisions. The reduced size of these incisions makes it possible to achieve a faster recovery rate, minimize the risk of infections and cause less pain compared to open surgery. These medical benefits have led to the widespread use of laparoscopy in numerous operations, becoming the standard for many organ systems, especially gynecological and digestive [1].

Unfortunately, laparoscopic operations are not easy to perform. The surgeon loses tactile feedback on the patient’s tissues as the clinician must manipulate them using long laparoscopic instruments. Furthermore, clinicians cannot perceive a three-dimensional scenario as in open surgery. Laparoscopic operations are always observed through a monitor that significantly restricts the field of vision and on which the surgeon completely loses the sensation of depth. These technical difficulties make any manipulation of tissues more complicated.

To facilitate clinicians’ work during surgeries, as well as allowing robots to collaborate with them in a wide range of tasks, intensive efforts have been made to improve automation and to increase surgeons’ capabilities [2]. A part of these efforts are focused on obtaining information from a laparoscopic video signal, where different tasks have been tackled, e.g., replacing physical sensors through the estimation of forces using images [3]; classifying actions such as the different steps of a suture procedure [4]; allowing collaborative robots to understand what actions the surgeon is carrying out; or evaluating different surgery-related skills [5,6,7,8].

Furthermore, multiple studies are centered on enhancing artificial perception of surgery-related elements, as localizing, detecting, and tracking these elements are crucial for robotic applications; for example, tracking surgical tools [9,10,11,12], which can be used in multiple tasks [13] such as surgery-video analysis based on tool presence [14,15] or classification of different surgical phases based on the tools that appear in the video signal [16]. Additionally, although many of the studies deal with 2D estimation, proposals working with 3D model estimates of surgery elements can also be found [17,18].

Although extensive work has been done in surgical tool detection using deep learning architectures, to the best of our knowledge, this has not been extended to surgical gauzes. Gauze detection algorithms not only allow for greater accuracy and reliability in the determination of the operation phase, but also enable the development of other more immediate applications. In not-robotized laparoscopic surgery, it is feasible to track the gauze by processing the captured video signal provided by the endoscope in a transparent and unattended way for the health personnel [19,20,21]. Automated gauze tracking relieves medical staff from routine counting and control tasks to avoid the inadvertent retention of these items, a medical error that occurs rarely, but it can cause serious complications in the patient’s health [22,23,24].

It seems clear that the detection and localization of gauze in the images from the endoscope can provide valuable information to automate numerous tasks in the operating room. However, gauze detection in images is not straightforward due to its variable appearance, and because, when they are soaked in blood or other body fluids, they blend with the patient’s internal tissues [21]. Furthermore, in surgical operations, the illumination inside the patient’s body is not uniform and it often generates frequent brightness on the organs. Additionally, the endoscopic camera is subject to movements that are not always smooth, and smoke can appear on the scene due to tissue cauterization. All these practical difficulties cause poor image quality due to saturation, blurring or defocusing.

In other circumstances, under steady imaging conditions, gauze texture features could be modelled explicitly with hand-crafted descriptors, expecting good results in classification. However, previous work in surgical gauze detection in images [19,20,21] shows that it is challenging to achieve good robustness with traditional methods for feature extraction in these complex situations and they always require a great deal of programming effort. In uncontrolled environments where imaging conditions fluctuate and when the intra-class variability of the features is important, it is known that Convolutional Neural Networks (CNNs) are more robust and deliver superior results than traditional descriptors [21,25] with the only shortcoming of their computational burden.

In this article, we present a gauze presence and location dataset [26] for training machine learning models, allowing the development of automated models to detect surgical gauze on endoscopic video signal, which provides key information to infer the stage of the operation, crucial in tasks such as in autonomous robotic assistants and temporal segmentation of laparoscopic videos into surgical phases. Alongside the gauze presence and location dataset for training models presented in this paper, we analyzed a set of baselines using well-established computer vision machine learning architectures that have been trained and tested using our data. These baselines are object detection using YOLOv3, coarse segmentation using various convolutional backbones, and semantic segmentation using a U-Net architecture. Special attention has been paid to runtime due to the need to process video frames in real time in most applications, such as in the case of autonomous robot guidance or in gauze tracking to avoid inadvertent retentions.

The rest of the document is organized as follows. In the following section, we introduce our dataset and the proposed train/evaluation distribution, the localization tasks which we present baselines for, and the architectures we have trained. Section 3 reports on the experimental results, with Section 4 focused on the discussion of those and the conclusions of the study.

## 2. Materials and Methods

In the following section, the main contribution of the paper, the dataset, is presented. Furthermore, we detail the different techniques used for the image analysis tasks, as well as the hardware and software used during the study, followed by the evaluation methodology.

### 2.1. Dataset

The presented dataset [26] consists of 42 video files (30 with gauze presence, ~33 min in total and 12 without gauze presence, ~13 min), collected in different settings. The videos were acquired using a STORZ TELECAM One-Chip Camera Head 20212030, color system PAL with integrated focal Zoom Lens, f = 25–50 mm (2×), with an image sensor of ½ inch CCD and 752 (H) × 582 (V) pixels per chip (PAL) equipped with a Hopkins telescope 0°, 10 mm, 31 cm.

**Figure 1:**
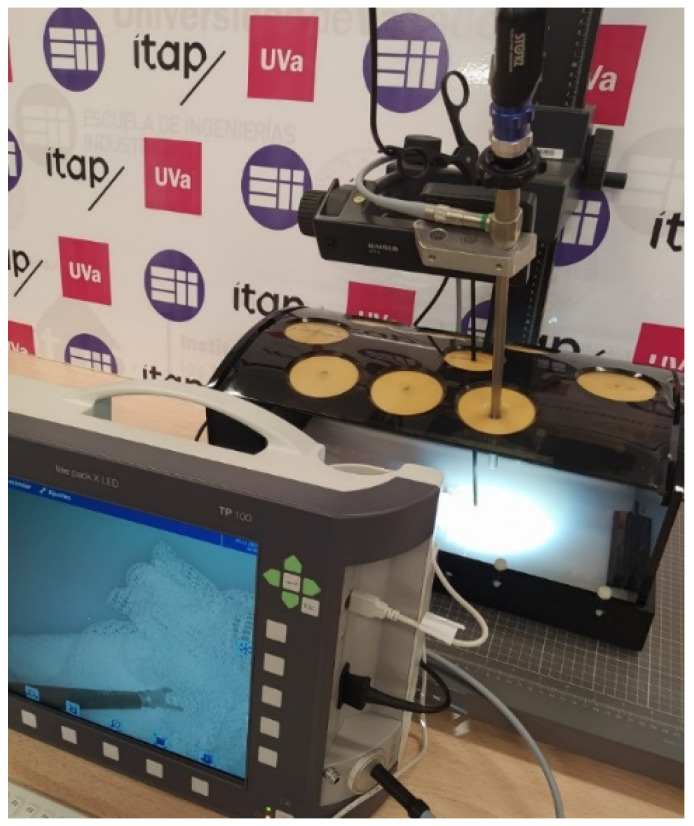
The videos have been recorded using a laparoscopic simulator with a Storz Telecam.

**Figure 2:**
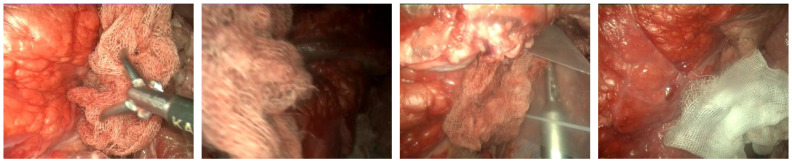
Different frames sampled from the dataset.

The videos have not been recorded in laparoscopic surgery operations on real patients. In order to test the gauze detection algorithm under the different conditions, diverse operation scenarios have been recreated in a laparoscopic simulator (Figure 1) using animal organs (No live animals have been used in our experiments. The pig organs used in our scenarios were provided by a certified local slaughterhouse). The scenarios include the following situations: absence of gauze, absence of gauze with tools presence, presence of a unique clean gauze and tools, presence of a unique stained gauze and tools, and presence of multiple gauzes in different states. Some frames from different videos are shown in Figure 2. An overview of the characteristics of each video, as well as the directory structure, are included in Appendix A.

**Figure 3:**
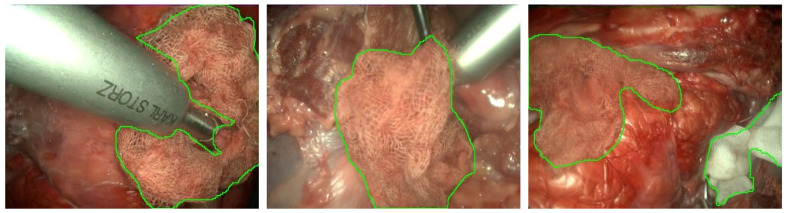
Colorized outline of gauze masks from the dataset over their correspondent frame. Best viewed in color.

A subset of these videos (18 out of the 30 videos with gauze) has been sampled every 10 frames, resulting in 4003 frames, where a binary mask for the gauze has been manually traced. Some samples of these generated masks are presented in Figure 3. This way, the dataset enables supervised training in semantic segmentation tasks.

The human-generated masks have been used to automatically generate and classify fragments of 100 × 100 pixels based on the presence or absence of gauze in the mask corresponding to each frame. This automatic generation results in 168,126 fragments (80,918 with gauze and 87,208 without), which are extensively used in our work and are therefore included in the presented dataset (As the fragments are generated and classified automatically based on the masks, it is trivial to generate them of any desired size. A Python script to do so is included in the dataset).

### 2.2. Train-Evaluation Split of the Dataset

To ensure independence between training and evaluation sets, as consecutive frames in a video may be too similar and pollute the evaluation set, random splits have been discarded. Instead, the dataset has been split video-wise in the following fashion (Table 1), ensuring equilibrium between tool presence, blood impregnation and movement of the telecam in both partitions. The resulting distribution of fragments and masks is presented in Table 2.

### 2.3. Gauze Detection

Object detection is the pillar of many computer vision applications ranging from face detection in security systems to pedestrian detection in autonomous vehicle driving. Detection techniques have undergone enormous development in the last five years thanks to the advance of convolutional neural networks. Given an image or video stream, object detection involves locating and classifying the searched objects present in the image. The outcome of a detection algorithm is usually represented by labelled bounding boxes marked on the items that appear in the image.

Region-based CNNs methods consider object detection as a two-stage problem: generating the region proposals and then classifying those regions. However, a few recent algorithms pose object detection from an integrated approach by feeding the input image into a single convolutional network that predicts the bounding boxes and their class probabilities. The YOLO (You Only Look Once) algorithm, proposed by Redmon et al. [27] in 2016, implemented a unified approach by posing object detection as a regression problem. YOLO is recognized as one of the most efficient algorithms, suitable for real-time processing, due to this evaluation with a single convolutional network.

In YOLO, the input image is initially divided into 13 × 13 square cells and then each cell predicts B = 2 bounding boxes. The size of these bounding boxes is preset from the ground truth in the data set. Each bounding box is associated with the probability that an object is contained inside the box (objectness score) and the probabilities of the detected object belonging to a particular class (class confidences).

The third version of the algorithm, YOLOv3 [28], increased its performance even more introducing an improved backbone classifier, Darknet-53. This backbone includes residual connections and has more layers than its predecessor (Darknet-19). YOLOv3 carries out the prediction at three different scales, downsampling the input image’s dimensions by a factor of 32, 16 and 8. This multiscale approach enhances YOLOv3 capacity to deal with different scales.

We have implemented this object detector as the authors propose in their paper [28], which includes Darknet-53 as a backbone classifier.

### 2.4. Gauze Coarse Segmentation

Gauze inside the patient’s body does not exhibit a defined shape like the tools used in surgery. The form that gauzes adopt in operations is unpredictable and variable. Its texture, on the other hand, is highly discriminating.

**Figure 4:**
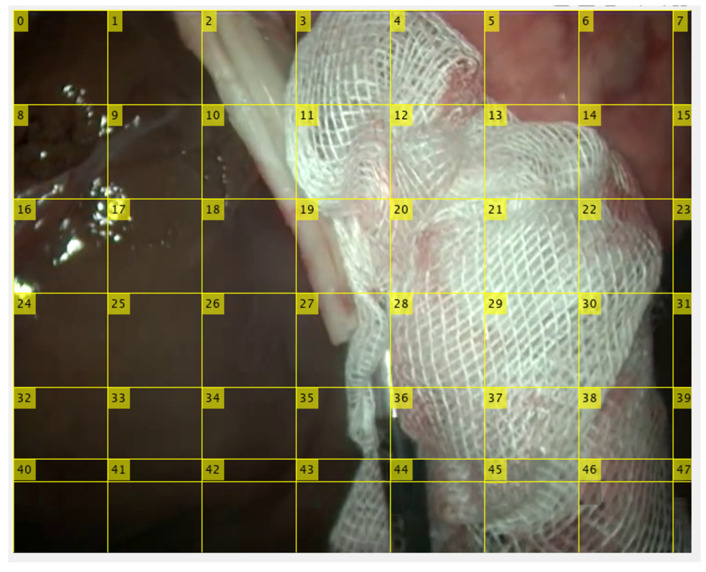
Each video frame is divided into square cells of size 100 × 100 to classify each cell as gauze or background. Cells on the left are discarded by its irrelevant dimensions and those in the last row are partially overlapped with the previous row to maintain the 100 × 100 size.

In order to mainly consider gauze texture, we propose a coarse segmentation approach that focuses on area analysis rather than on shape analysis. For this reason, in this second approach, the image has been divided into square cells (Figure 4) to categorize each one individually as gauze or background. This approach is not feasible in the case of a general detector because it is always required that the entire object appears in the analyzed window. However, in the case of gauze, the analysis of a fragment allows a reliable classification due to its particular texture.

Some tests have been carried out to establish the most appropriate size of the image cells. A priori, the use of small cells would seem to be the right option because they would allow to define more precisely the gauze in the image. However, the poor results obtained in the tests indicate that classifying very small cells is not a good option, probably because their low area makes them statistically more unstable in the classification. On the other hand, if the size is too large, the area of analysis is more significant, but the detection is subject to the gauze occupying a good part of the cell, which can be difficult when cell dimensions are excessive. Tests have suggested that a cell size of 100 × 100 pixels is a good compromise between these two situations.

However, as the size of the images provided by the endoscope is 720 × 576, smaller cells will be generated at the left and lower edges of the image. Cells on the right edge (cells number 7, 15, …, 47 in Figure 4) will not be processed due to their irrelevant size (20 × 100). However, the last row of the image (cells number 40 to 46 in Figure 4) will be considered because they are more significant. In this case, the 100 × 100 areas have been sampled from the bottom edge. This generates a small overlap of 100 × 24 with the squares of the previous row but allows to preserve the square shape of cells.

Classification of cells in the image have been solved with CNNs through transfer learning. We have analyzed the performance in gauze detection using three different CNN models: InceptionV3 [29], MobileNetV2 [30] and ResNet-50 [31]. All these architectures are formed by a set of convolutional layers that extract features from the images progressively, this is, starting with low-level features and transitioning into high-level features as the input advances through the network layers. However, each one of the mentioned networks is characterized by different additional mechanisms, such as inception modules in InceptionV3, depthwise and pointwise convolutions along linear bottlenecks in MobileNetV2, or residual connections in the ResNet-50 model.

In order to perform binary classifications, every model last layer has been modified to have only one output neuron. Also, image fragments have been scaled to each model input size. The number of layers and parameters, as well as the input size in each of these networks, is summarized in Table 3.

### 2.5. Gauze Segmentation

Some applications such as robotic manipulation, however, require an as-exact-as-possible shape detection solution. Given an input image, either static or from a video frame, semantic segmentation architectures label each pixel in said image, classifying them in one of the possible classes. Supervised segmentation approaches are usually difficult to train, as non-synthetic ground truth is scarce and difficult to obtain, therefore the value of the hand-traced masks of gauzes in our dataset [26] for this type of task.

Along the past years, multiple semantic-segmentation-oriented approaches have been proposed using fully convolutional networks [32] models based on regional convolutional networks (Mask R-CNN [33] which extends Faster R-CNN [34], encoder–decoder-based models such as SegNet [35], and dilated convolutions as in DeepLab publications [36,37].

In this work, we present a baseline for gauze semantic segmentation using a U-Net-based architecture (implementation from [38]). This architecture is formed by an encoding block, where each layer extracts and downsamples feature maps from the previous input to pass them to the next layer, and a decoder block where each layer fuses the corresponding representation from the encoder and the previous layer upsampled output to generate the segmentation map. The encoder in our study has an unfrozen MobileNetV2 [39] pretrained on the 2012 ILSVRC ImageNet dataset [40] as the feature extractor and a decoder with blocks of {256, 128, 64, 32, 16} kernels. During training, an Adam [41] optimizer was used with a learning rate of 0.0001, and, following the original Adam paper proposal, β1, β2 and ε values of 0.9, 0.999 and 1 × 10^−7^, respectively. The optimized function has been Dice Loss [42]. Furthermore, as input image size to the network is 320 × 320, a bilinear interpolation resizing operation is incorporated in the preprocessing of the image, allowing training and inference on original size images.

Due to the nature of the task, segmentation training can be improved if the dataset is augmented to present image flips, scale variations, rotations, and changes to the brightness and sharpness of the image. The intuition behind these transformations is that both gauzes and the laparoscopic telecam are constantly changing orientation and position, hence the geometric transformations. Blurriness due to stains in the lens and movement is also common, which motivates the application of Gaussian blur, sharpness filters, and movement blur augmentations. Ultimately, as light coming from the telecam can also affect the appearance of gauze and surgical instruments, contrast and brightness augmentations are also considered. This set of transformations is randomly parametrized and applied each epoch, increasing the number of relevant images and reducing the variance of the trained model. The final model was obtained training on the augmented dataset for 10 epochs.

### 2.6. Hardware and Software

The models presented in this paper are public-domain implementations of well-established models, mentioned in their corresponding sections, which have been trained and tested using Python 3.7 (Python Software Foundation, Wilmington, NC, USA) and the deep learning framework Keras 2.6 (Various authors, no location). Image pre-processing and labelling have been carried out using the OpenCV 4.5 (OpenCV, Palo Alto, CA, USA) Python package. Training (fine tuning) and test inference of all models have been carried out using a system with the following specifications (Table 4).

### 2.7. Evaluation

Regarding the evaluation of the performance of the methods presented, based on the task the model takes on, different metrics such as Intersection over Union (IoU), accuracy, precision, recall, F1 Score, and the frames processed per second (FPS) have been computed.

Intersection over Union (IoU) measures the relation between overlapped area in the ground truth and predicted mask or bounding box and area of the union of said elements. In this work, we consider binary masks for semantic segmentation.

(1) $$\mathrm{IoU}=\frac{\mathrm{Area}\;\mathrm{of}\;\mathrm{Overlap}}{\mathrm{Area}\;\mathrm{of}\;\mathrm{Union}}\;,$$

Accuracy is the most intuitive evaluation of performance, and it is simply the relation between the image cells that are correctly classified to the total of cells:

(2) $$\mathrm{Accuracy}=\frac{\mathrm{TP}+\mathrm{TN}}{\mathrm{TP}+\mathrm{FP}+\mathrm{FN}+\mathrm{TN}}\;,$$

where

TP: true positives, image cells correctly marked as gauze.

TN: true negatives, cells correctly marked as background.

FP: false positives, cells wrongly marked as gauze.

FN: false negatives, cells wrongly marked as background.

Precision estimates what fraction of image cells classified as gauze are correct. It is defined as the number of fragments correctly marked as gauze (TP) divided by the overall number of them marked as gauze (TP + FP):

(3) $$\mathrm{Precision}=\frac{\mathrm{TP}}{\mathrm{TP}+\mathrm{FP}}\;,$$

The recall or sensitivity refers to the fraction of the image cells with gauze that are detected:

(4) $$\mathrm{Recall}\;\left(\mathrm{Sensitivity}\right)=\frac{\mathrm{TP}}{\mathrm{TP}+\mathrm{FN}}\;,$$

The proper algorithm should have a balance between precision and sensitivity. This means, in our case, that the algorithm should detect all the cells in the image where gauze is present and should not misclassify as gauze the samples corresponding to the background. The F1 Score combines the precision and recall of a classifier into a single metric. The value of the F1 Score ranges from zero, if either the precision or the recall is zero, to one, for a classifier with perfect precision and recall.

(5) $$F1\;\mathrm{Score}=\frac{2}{\frac{1}{\mathrm{precision}}+\frac{1}{\mathrm{recall}}}\;,$$

Another balanced metric is the Matthews Correlation coefficient (MCC), which takes into account the ratios of every category in the confusion matrix.

(6) $$\mathrm{MCC}=\frac{\mathrm{TP}\;\times \;\mathrm{TN}-\mathrm{FP}\;\times \mathrm{FN}}{\sqrt{\left(\mathrm{TP}+\mathrm{FP}\right)\left(\mathrm{TP}+\mathrm{FN}\right)\left(\mathrm{TN}+\mathrm{FP}\right)\left(\mathrm{TN}+\mathrm{FN}\right)}}\;,$$

Average precision (AP) estimates the average precision value for recall value over 0 to 1, this means the area under the precision-recall curve.

(7) $$\mathrm{AP}=\int_{0}^{1}p\left(r\right)\mathrm{dr}\;,$$

Mean average precision (mAP) is the average AP for all the classes, this metric measures the net’s accuracy doing a location and classification of the object.

Finally, the speed of each network, in Frames Per Second (FPS), has been calculated as the average amount of frames of video the CNN can process per second.

## 3. Results

In this third section, both quantitative and qualitative results from the models trained for the three different tasks on the proposed dataset are presented.

### 3.1. Gauze Detection

Gauze inside a patient´s body can have a large variety of shapes and colors due to being a deformable object impregnated with a patient´s blood. Thanks to multiscaling at three different scales, YOLOv3 can detect gauzes at higher speeds and precision than other CNN methods. For gauze detection, the same clusters as Redmon paper [33] have been used to determine bounding box prior: (10 × 13), (16 × 30), (33 × 23), (30 × 61), (62 × 45), (59 × 119), (116 × 90), (156 × 198), (373 × 326).

To evaluate the performance of YOLOv3, Redmon [28] uses mAP obtaining 57.9%. Table 5 shows the results obtained with YOLOv3 on our dataset [26]. A higher mAP has been achieved in our case, because the existence of a single class in the training facilitates better results than when many classes are present. In addition to mAP, other metrics such as Precision, Recall, F1 Score and FPS are presented in Table 5 to establish comparisons between the different methods of gauze detection used in this paper. Regarding execution time, YOLOv3 exceeds 30 FPS, thus allowing real-time detection of gauze.

**Figure 5:**
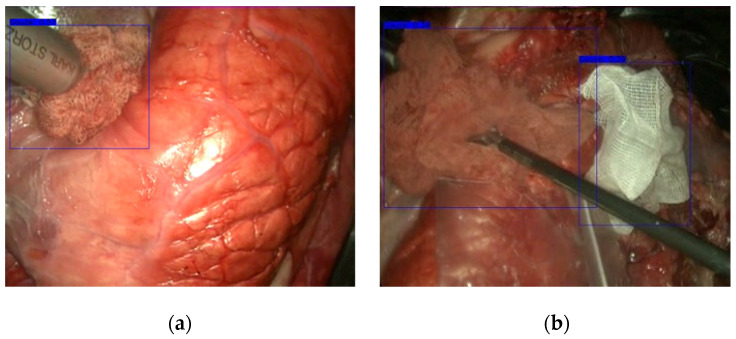
Results obtained with YOLOv3 on (**a**) blood-stained gauze and (**b**) both clean and blood-stained gauzes in the same image.

Figure 5 shows a frame with a blood-stained gauze detection where it can be seen an accurate and perfectly contained gauze, even though the background had huge similarities with it.

**Figure 6:**
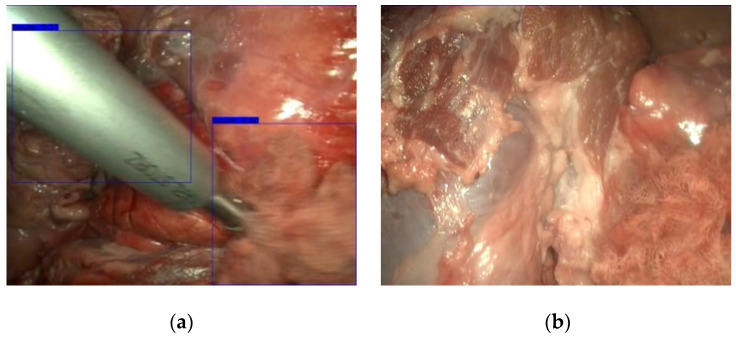
YOLOv3 detection errors. (**a**) The surgical tool has been marked as gauze due to a large glint on its metallic surface. (**b**) Gauze in the lower right corner has been overlooked.

As shown in Figure 5, YOLOv3 achieved a robust detection, being able to accurately label the gauze. DarkNet backbone can detect both clean and blood-stained gauzes, but the detection is better on clean gauze. However, the detection is not always perfect. Figure 6 shows two failures of YOLOv3. In the first one, although the gauze present in the image is detected, the surgical instrument is also marked, because the huge glint in this large metal tool confuses the algorithm. In Figure 6b, the gauze in the lower right corner of the image has been overlooked because, in this case, the gauze appears completely blended with the surrounding tissues.

### 3.2. Gauze Coarse Segmentation

In this section, we present the results of the coarse segmentation with InceptionV3, MobileNetV2 and ResNet-50. ResNet-50 has achieved the highest result with 94.09% average precision, followed by MobileNetV2 with 84.23% and InceptionV3 with 75.67%. Training and validation of the three networks have been carried out using the videos in Table 1.

Table 6 summarizes the overall results of precision, recall, F1 Score, Matthews Correlation Coefficient, and FPS that have been obtained with the InceptionV3, MobileNetV2 and ResNet-50 backbones. Regarding the F1 Score, ResNet-50 has reached a 92.67% score, making it the most suitable option for best results in gauze coarse segmentation.

In terms of processing time, InceptionV3 has shown a rate of around 21 FPS. This speed is about half the rate achieved by YOLOv3. ResNet-50 is the slowest with 13.70 FPS, followed by MobileNetV2 with 18.77 FPS. Although the three neural networks have shown decent results in terms of accuracy and sensitivity, InceptionV3 seems to be the best option when fast execution speed is needed doing coarse segmentation.

**Figure 7:**
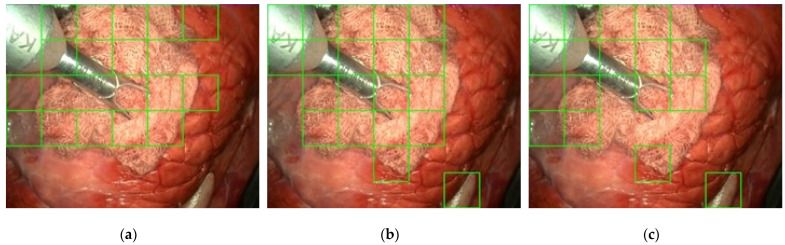
Results obtained using (**a**) ResNet-50, (**b**) MobileNetV2 and (**c**) InceptionV3. Green squares denote the fragments that have been classified as gauze.

Figure 7 shows the results obtained on the same frame using the three pretrained networks. The most robust neural network is ResNet-50, whose accuracy and recall were around 90%. However, the FPS rate was lower compared to the rest. Figure 7a shows that the detection was successful. Also, on this randomly selected frame, MobileNetV2 (Figure 7b) has perfectly detected the gauze cells in the image. The main difference is that MobileNetV2 has included one additional square in the lower part of the image corresponding to a false positive. Nonetheless, the endoscope has been fully detected as a gauze by this network. On the other side, InceptionV3 (Figure 7c) has both missed gauze fragments and erroneously marked the area in the lower part of the image as gauze, probably misled by the similar texture of this area.

### 3.3. Gauze Segmentation

In this last results section, we present the results of the U-Net architecture for semantic segmentation on the previously defined evaluation partition of the dataset. Metrics from this evaluation are presented in Table 7.

Segmentation of the complete image yields outstanding results, even when compared to those of both YOLOv3 and the patch-based classification approaches in terms of detection, while the use of MobileNetV2 as a backbone allows fast inference up to real-time speed (+30 FPS).

**Figure 8:**
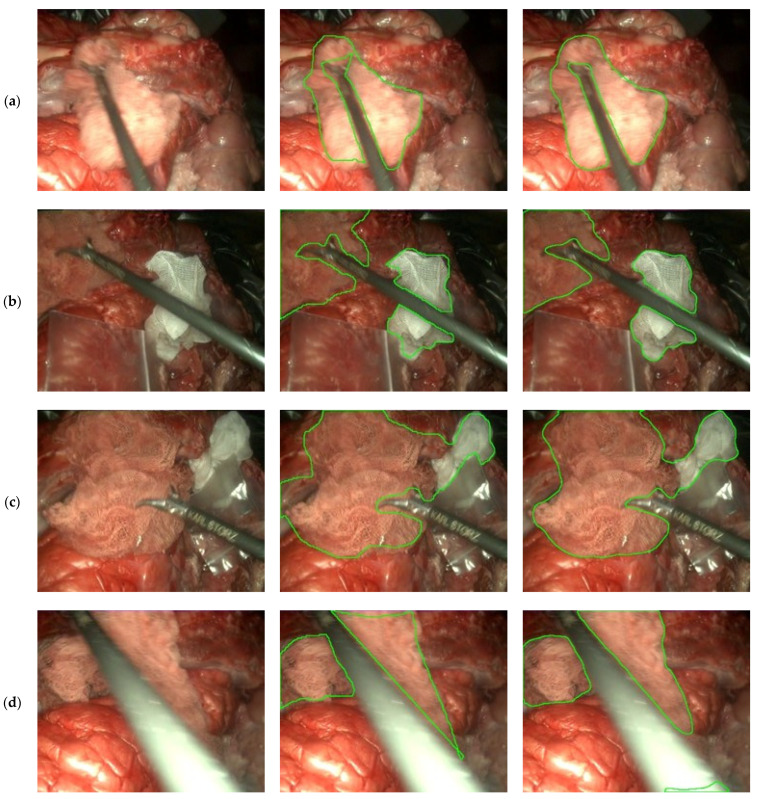
Segmentation results on the evaluation set. **Left** column: Input image, **center** column: hand-labeled ground truth, **right** column: U-Net prediction. Each row (**a**–**d**) corresponds to a different frame from the evaluation videos. Best viewed in color.

Figure 8 shows a set of results obtained with this model. In these results, the model capacity to segment surgical instruments out of the gaze mask (Figure 8a,b) is shown, as well as the ability to detect both clean and soaked gauzes, even when no discontinuation exists between them (Figure 8b,c). Lastly, in Figure 8d, a miss-detection of the gauze due to notable instrument movement blurriness and brightness appears in the lower-right corner of the image with the predicted mask (right column).

## 4. Discussion

The detection of surgical tools in laparoscopic video using CNNs is a very active area of research. However, gauze detection and localization has been almost unexplored even though these techniques can provide a considerable amount of supplementary information to automatize surgical applications. The variable appearance of gauze and their tendency to blend with a patient’s tissues may have prevented the development of detection algorithms. Although several datasets have been proposed in the context of laparoscopic surgery, these datasets focus on surgical tools, both presence detection [43] and segmentation [44,45] also covering anatomical [46,47] and surgical actions [46,47,48]. To the best of our knowledge, no dataset labelling gauzes has been published. The lack of a dataset with ground truth annotations of gauzes during a surgical procedure hinders the research of new approaches to this problem. Our dataset [26] amends this, with hand-labelled images of gauzes in different situations and environments.

In respect of the results obtained from the proposed baselines, the YOLOv3 architecture underperforms when compared with the other two methods with a recall of around 76%; however, it can process images at more than 30 FPS. This limitation in results quality is probably due to the virtually unlimited number of shapes the gauzes can take, which conflicts with the limited predefined anchors of these models.

The coarse segmentation approach, which is patch-based and has been tested with different backbones, presents satisfactory results (accuracy of around 90% using ResNet-50) when considering that no pre-processing has been applied to the resulting patches (e.g., thresholding the number of patches to eliminate outliers, or taking into consideration neighbor patches classification to ensure consistency). However, this approach lacks inference speed, mainly because an actual frame is composed of 42 patches, and therefore, an inference speed of 30 FPS with this method requires the classification of 1260 images every second by the entire network, which limits the execution of these models at real-time speeds.

In the U-Net segmentation baseline, using a MobileNetV2 architecture has resulted in a good compromise between inference speed and results quality, as it is possible to execute the model at real-time speeds (above 30 FPS) on a video signal while obtaining an IoU of more than 0.85 on the defined evaluation set of the dataset. The accuracy reached by U-Net in gauze segmentation and its speed of execution make this architecture perfectly suited for real-time applications such as gauze manipulation in autonomous robots. The errors visualized on these results come mainly from movement blur in anatomical structures and extreme brightness in surgical tools. To remediate this, a model trained on the previously mentioned datasets containing tools and anatomic information would relieve false gauze positives since the system could identify tools as the correct object.

Finally, as the proposed dataset [26] enables the development of novel architectures and applications related to laparoscopic surgery, further work englobes multiple paths such as the training of transformers architectures [49,50] for different tasks, as these have been proven to present outstanding results in various domains, the development of temporal sequence models that take into account past frames to enhance the results, or, as previously mentioned, the incorporation of gauze detection into a laparoscopic surgery segmentation framework capable of complete scene segmentation.

In conclusion, these results are encouraging and demonstrate that the dataset proposed in this paper enables accurate gauze detection and segmentation using CNNs, even in real time, paving the way for the development of new practical applications such as enhancing perception of autonomous surgical robots, making them capable of tracking and manipulating gauzes in real time to manipulate during different surgical procedures; not-robotized minimally invasive surgery applications like gauze tracking in the endoscope video to avoid inadvertent gauze retention, serving as an additional tool for surgery nurses during laparoscopic procedures; or cataloging tasks, such as offline scene labelling and video indexing to separate different stages of surgeries, which facilitates multimedia querying by students and professionals.

## Figures and Tables

**Table 1:** Video-wise train and evaluation splits.

Split	Videos
Train	VID00 {02, 03, 06, 07, 10, 13, 17, 18, 22, 23, 25, 30}
Evaluation	VID00 {04, 11, 16, 21, 24, 28}

**Table 2:** Distribution of fragments and masks in each split.

Split	Fragments		Masks
	Gauze	No Gauze	
Train	61,860 (76.45%)	64,938 (74.46%)	3019 (75.42%)
Evaluation	19,058 (23.55%)	22,270 (25.54%)	984 (24.58%)

**Table 3:** Summary of the tested CNN architectures.

Network	Layers	Parameters (Millions)	Input Size (Pixels)
InceptionV3	48	23.8	299 × 299
MobileNetV2	28	2.5	224 × 224
ResNet-50	50	25.6	224 × 224

**Table 4:** Technical specifications of the computing system.

Hardware	Model
Processor	AMD Ryzen 7 3800X
Memory	DDR4 16GB × 2 (3000 MHz)
Graphics Processing Unit	NVIDIA GeForce RTX 3070, 8GB GDDR6 VRAM
Operating System	Ubuntu 20.0 LTS 64 bits

**Table 5:** Overall results obtained using the evaluation videos.

Network	Precision [%]	Recall [%]	F1 Score [%]	mAP [%]	FPS
DarkNet-53	94.34	76.00	84.18	74.61	34.94

**Table 6:** Overall results provided by the pretrained networks in the evaluation videos.

Network	Precision [%]	Accuracy [%]	Recall [%]	F1 Score [%]	MCC [%]	FPS
InceptionV3	75.67	77.68	89.08	81.82	54.60	21.78
MobileNetV2	84.23	75.67	76.68	80.28	49.08	18.77
ResNet-50	94.09	90.16	91.31	92.67	77.76	13.70

**Table 7:** Results of the U-Net model in the evaluation videos.

Model	IoU	Frames per Second (FPS)
U-Net	0.8531	31.88

## Data Availability

The data presented in this study are openly available in the Zenodo data repository at https://doi.org/10.5281/zenodo.6637871 (accessed on 13 June 2022).

## Appendix A

This appendix overviews the characteristics of each video in the proposed dataset. “Gauzes” indicates the maximum number of gauzes in any frame of the video, “Stained” describes if the gauze(s) have blood in them, “Objects” indicates the presence of objects other than gauzes in the video and, lastly, “Camera” specifies if the laparoscope was moving during the recording of the video.

**Table A1:** Overview of the characteristics of each video.

Video	Gauzes	Stained	Objects	Camera	Duration
VID0002	1	Yes	Tools (1)	Movement	0 min 49 s
VID0003	1	Yes	Tools (1)	Movement	1 min 3 s
VID0004	1	Yes	Tools (1)	Movement	1 min 7 s
VID0005	0	-	None	Movement	1 min 7 s
VID0006	1	Yes	Tools (1)	Movement	1 min 40 s
VID0007	1	Yes	Tools (1), Plastic bag	Movement	2 min 45 s
VID0008	0	-	Tools (1)	Movement	2 min 43 s
VID0009	0	-	None	Movement	0 min 23 s
VID0010	1	Yes	Tools (1)	Movement	1 min 42 s
VID0011	1	Yes	Tools (1)	Movement	1 min 15 s
VID0012	0	-	Tools (1)	Movement	0 min 54 s
VID0013	1	Yes	Tools (1)	Movement	0 min 32 s
VID0014	0	-	Tools (2)	Movement	1 min 19 s
VID0015	0	-	Tools (2)	Movement	0 min 44 s
VID0016	1	Yes	Tools (2)	Static	1 min 1 s
VID0017	1	Yes	Tools (1)	Static	0 min 50 s
VID0018	1	Yes	Tools (2), Plastic bag	Static	0 min 20 s
VID0019	0	-	Tools (1), Plastic bag	Static	0 min 52 s
VID0020	0	-	None	Movement	0 min 25 s
VID0021	1	Yes	Tools (1)	Movement	0 min 41 s
VID0022	1	No	Tools (1)	Movement	1 min 28 s
VID0023	2	Both	Tools (1)	Movement	2 min 47 s
VID0024	2	Both	Tools (1), Plastic bag	Movement	1 min 40 s
VID0025	2	Both	Tools (1), Plastic bag	Movement	1 min 1 s
VID0026	0	-	None	Movement	0 min 11 s
VID0027	0	-	Tools (1)	Movement	2 min 53 s
VID0028	2	Both	Tools (1)	Static	0 min 48 s
VID0029	0	-	Tools (1)	Movement	0 min 48 s
VID0030	2	Both	Tools (1)	Movement	5 min 7 s
VID0031	0	-	None	Movement	0 min 33 s
VID0100	1	No	Tools (1)	Static	0 min 38 s
VID0101	1	No	Tools (1)	Static	0 min 33 s
VID0102	1	No	Tools (1)	Static	0 min 43 s
VID0103	1	Yes	Tools (1)	Static	0 min 28 s
VID0104	1	Yes	Tools (1)	Movement	0 min 39 s
VID0105	1	Yes	Tools (1)	Movement	0 min 39 s
VID0106	1	Yes	Tools (1)	Static	0 min 24 s
VID0107	1	Yes	Tools (1)	Static	0 min 27 s
VID0108	1	Yes	Tools (1)	Static	0 min 32 s
VID0110	1	Both	Tools (1)	Static	0 min 51 s
VID0111	1	Yes	Tools (1)	Static	0 min 27 s
VID0112	1	Yes	Tools (1)	Static	0 min 19 s
